# Supplementary material for: Neonatal Morbidities and Feeding Tolerance Outcomes in Very Preterm Infants, before and after Introduction of Probiotic Supplementation
Source: Nutrients. 2022 Sep 3;14(17):3646. doi: 10.3390/nu14173646 (PMC9460632; doi:10.3390/nu14173646)
Supplement: Supplementary file 1 [file nutrients-14-03646-s001.zip › nutrients-1824914-supplementary.pdf]

Table S1. Characteristics of 180 very preterm infants born in Stockholm, of which 139 received probiotics per the new guideline while 41 infants were eligible but were not supplemented.

	No probiotics (*n* = 41)	Probiotics (*n* = 139)	p-value
**Perinatal characteristics**[a]			
Gestational age, weeks (SD)	30.5 (1.0)	30.2 (1.1)	0.10
Birth weight, Fenton Z-score	−0.06 (0.9)	0.02 (0.8)	0.70
Birth weight for gestational age, n (%)			
Appropriate	36 (87.8)	122 (87.8)	0.92
Large	1 (2.4)	5 (3.6)	
Small	4 (9.8)	12 (8.6)	
Male, n (%)	22 (53.7)	68 (48.9)	0.59
Singleton, n (%)	34 (82.9)	100 (71.9)	0.16
Vaginal delivery, n (%)	9 (21.9)	42 (30)	0.34
Apgar score < 7 at 5 minutes[b], n (%)	8 (20.5)	18 (13.0)	0.25
Transient tachypnea, n (%)	16 (39.0)	60 (43.2)	0.64
RDS, n (%)	20 (48.7)	56 (40.3)	0.33
Invasive ventilation[c], days (SD)	2.7 (3.6)	2.4 (2.6)	0.82
CPAP/HFNC[d], days (SD)	12.6 (12.1)	13.4 (14.6)	0.78
BPD, n (%)	1 (2.4)	4 (2.9)	0.88
PDA, n (%)	0 (0.0)	4 (2.9)	0.27

[a] presented as mean value and standard deviation, or number of infants and proportion
[b] missing data *n* = 3
[c] based on 9 and 19 infants needing any invasive ventilation in the No probiotics and Probiotics groups, respectively
[d] based on 37 and 128 infants needing CPAP and/or HFNC in the No probiotics and Probiotics groups, respectively

Table S2. Rates and risks of the composite outcome of death, sepsis, and/or surgical NEC, in 345 very preterm infants

	No probiotics (*N* = 206)	Probiotics (*N* = 139)	Crude RR [95% CI]	Adjusted RR[a] [95% CI]
Composite outcome of death, sepsis and/or surgical NEC, n (%)	16 (7.8)	5 (3.6)	0.46 [0.17 – 1.23]	0.44 [0.17 – 1.15]
Single outcomes of death, sepsis or surgical NEC, n (%)				
- Death	2 (1.0)	0 (0)	-	-
- Sepsis	15 (7.2)	5 (3.6)	0.49 [0.18 – 1.33]	0.46 [0.17 – 1.23]
- Surgical NEC	3 (1.5)	1 (0.7)	0.49 [0.05 – 4.70]	0.48 [0.05 – 4.53]

[a] risks adjusted for gestational age (days) and birth weight (Fenton Z-score)
